# Interleukin-10 haplotypes in Celiac Disease in the Spanish population

**DOI:** 10.1186/1471-2350-7-32

**Published:** 2006-03-31

**Authors:** Concepción Núñez, Diana Alecsandru, Jezabel Varadé, Isabel Polanco, Carlos Maluenda, Miguel Fernández-Arquero, Emilio G de la Concha, Elena Urcelay, Alfonso Martínez

**Affiliations:** 1 Department of Clinical Immunology, Hospital Clínico San Carlos, Madrid, Spain; 2 Department of Paediatric Gastroenterology, Hospital La Paz, Madrid, Spain; 3 Department of Paediatrics, Hospital Clínico San Carlos, Madrid, Spain

## Abstract

**Background:**

Celiac disease (CD) is a chronic disorder characterized by a pathological inflammatory response after exposure to gluten in genetically susceptible individuals. The HLA complex accounts for less than half of the genetic component of the disease, and additional genes must be implicated. Interleukin-10 (IL-10) is an important regulator of mucosal immunity, and several reports have described alterations of IL-10 levels in celiac patients. The *IL-10 *gene is located on chromosome 1, and its promoter carries several single nucleotide polymorphisms (SNPs) and microsatellites which have been associated to production levels. Our aim was to study the role of those polymorphisms in susceptibility to CD in our population.

**Methods:**

A case-control and a familial study were performed. Positions -1082, -819 and -592 of the *IL-10 *promoter were typed by TaqMan and allele specific PCR. IL10R and IL10G microsatellites were amplified with labelled primers, and they were subsequently run on an automatic sequencer. In this study 446 patients and 573 controls were included, all of them white Spaniards. Extended haplotypes encompassing microsatellites and SNPs were obtained in families and estimated in controls by the Expectation-Maximization algorithm.

**Results:**

No significant associations after Bonferroni correction were observed in the SNPs or any of the microsatellites. Stratification by HLA-DQ2 (DQA1*0501-DQB1*02) status did not alter the results. When extended haplotypes were analyzed, no differences were apparent either.

**Conclusion:**

The *IL-10 *polymorphisms studied are not associated with celiac disease. Our data suggest that the IL-10 alteration seen in patients may be more consequence than cause of the disease.

## Background

Celiac disease (CD) is a chronic inflammatory disorder characterized by a pathological response after exposure to gluten in genetically susceptible individuals [1]. HLA class II genes, mainly the alleles DQA1*0501 and DQB1*02 (HLA-DQ2) but also DQA1*0301 and DQB1*0302 (HLA-DQ8), have been found to play an important role in the development of celiac disease. However, HLA genes do not explain the totality of CD cases detected and other genes must be involved in CD onset. In fact, the sibling familial risk attributed to HLA is approximately half of the sibling risk for CD [2].

The histological lesions typical of CD seem to be associated with a predominant T helper type 1 (Th1) cell response. Thus, cytokines characteristic of a Th1 inflammatory response as interferon gamma, are produced by *lamina propria *T cells isolated from the intestine of CD patients after gluten stimulation [3]. The complex homeostatic regulation of immune processes involves numerous interacting cells and molecules. The prototypic regulatory cytokine in the intestinal mucosa is IL-10. Among other functions, its role as an inhibitor of Th1 cell development should be highlighted in the context of CD. Therefore, basal underproduction of IL-10 could eventually contribute to an exacerbated Th1 inflammation and this, in turn, might increase CD susceptibility. In fact, low levels of IL-10 have been found associated with different typical features of celiac disease such as anti-tissue transglutaminase antibodies [4] and studies using recombinant human IL-10 (rhIL-10) have shown a suppression of gliadin-specific T cell activation [5]. IL-10 knock-out mice develop chronic enterocolitis, and recent trials have investigated the possibility of using IL-10 as therapy in ulcerative colitis patients [6], another chronic inflammatory disease of the intestinal mucosa.

The *IL-10 *gene is located on human chromosome 1 (1q31-q32) and several polymorphisms have been described in its regulatory region. The *IL-10 *promoter contains single nucleotide polymorphisms (SNPs) in the proximal and distal regions. The proximal SNPs are at position -1082 (A/G), -819 (T/C) and -592 (A/C) and the strong linkage disequilibrium they display makes them appear in only three haplotypes (ACC, ATA and GCC) out of the theoretical maximum of eight. These variants have been reportedly involved in the transcription rate of *IL-10 *and therefore in the production level of this cytokine. Two microsatellites have been also described in the 5'-flanking region, IL10G and IL10R, at 1.1 and 4 kb from the origin of transcription, respectively. It has been described that haplotypes formed by those microsatellites are associated with different levels of IL-10 production [7]. The study of those polymorphisms seems then appropriate to study the possible implication of the *IL-10 *gene in CD susceptibility.

Several studies have been previously performed studying IL-10 as a candidate gene for different autoimmune or inflammatory diseases and some associations with specific alleles have been reported. For example, the allele 12 of the IL10G microsatellite was described as associated with multiple sclerosis [8] and rheumatoid arthritis [9], and a similar trend was shown in type 1 diabetes [10]; in Crohn's disease, association with IL-10G 14 and -1082G was reported [11]. In another study, [12] no association of IBD and proximal SNPs of IL-10 was observed, but microsatellites were not included.

A previous family-based study performed in Finnish population failed to show association between IL-10 polymorphisms and CD, but they studied only the promoter SNPs and not the IL10G and IL10R microsatellites [13]. A case-control study in Italian population again showed negative results, but only the -1082 (A/G) promoter SNP was analyzed in their samples [14].

Our aim was to perform a more comprehensive study including both SNPs and microsatellites, and perform a case-control and a family TDT analysis, in a large group of Spanish CD patients and controls.

## Methods

### Subjects

A total of 446 celiac disease (CD) patients (59% women) and 573 ethnically matched healthy controls (51% women) recruited from the Hospital Clínico San Carlos (Madrid, Spain) were studied to assess the influence of *IL-10 *polymorphisms in the predisposition to CD. We included parents (only one mother showed CD) of 189 patients (these were part of the above-mentioned 446 CD patients). Fourteen of the 446 CD patients also showed IgA deficiency. Celiac patients were diagnosed following the European Society for Paediatric Gastroenterology and Nutrition (ESPGAN) criteria [15]. All the samples were obtained from Spanish white individuals after obtaining their informed consent.

### Genotyping

CD patients were genotyped for two single nucleotide polymorphisms (SNPs), -1082 (G/A) and -592 (C/A), both located in the proximal promoter of the *IL-10 *gene. The study was performed by using TaqMan probes (C___1747360_10 for -1082 and C___1747363_10 for -592) under conditions recommended by the manufacturer (Applied Biosystems, Norwalk, USA). The samples were read by two observers and some samples with known results were included in the analysis as controls. Those two SNPs had been analyzed in controls using allele-specific PCR as previously described [8]. In the control group, a third promoter polymorphism, -819 (C/T), located between the other two SNPs, was also studied. This third SNP was found to be in complete linkage disequilibrium with the -1082 and -592 SNPs, and therefore it could be always deduced in CD patients. This intermediate position is written between parentheses in the present paper to remember that it was not really typed in all samples. The equivalence between the typing performed by those two different techniques was tested in 96 samples and no discrepancies were found. Two CA-repeat microsatellites in the 5'-flanking region of the *IL-10 *gene, designed as IL10R and IL10G, were also studied in CD patients and controls as previously described. In brief, a PCR is performed with labelled primers, and the products were electrophoretically run on an AbiPrism 3100 automatic sequencer (Applied Biosystems) [8]. HLA-DR and HLA-DQ typing was also performed as described before [16].

### Statistical analysis

Carrier, allelic and haplotypic frequencies in patients and controls were compared by means of chi-square tests and Fisher's exact test when necessary (expected values below 5), using the statistical package EpiInfo v5.00 (CDC, Atlanta, USA). The sample size in the present study would allow us to detect an OR value of 1.5 for an allelic frequency of 0.1 (at a significance level of 0.05) with a statistical power, calculated using the program found online [17], of 80%. Haplotypes were obtained by segregation analysis in the group of CD patients with families and they were instead estimated in the control group with the Expectation-Maximization (EM) algorithm implemented in the Arlequin program v2.000. Transmitted and non-transmitted haplotypes were compared using the Transmission Disequilibrium Test (TDT).

## Results

The case-control comparison of the carrier frequencies of the different alleles found in each of the two microsatellites studied, IL10R and IL10G, did not show any significant difference. Table 1 shows those values for the most frequent alleles of each microsatellite. In one case (allele 13 of IL10G), one p-value under 0.05 was found but the significance was lost when Bonferroni (9 comparisons) correction was applied. The study of the promoter SNPs showed no significant results either. The three haplotypes formed by those three SNPs, A(C)C, A(T)A and G(C)C were not differentially distributed when patients and controls were compared (Table 2).

**Table 1:** Carrier frequencies in IL-10R and IL-10G microsatellites in CD patients (n = 446) and controls (n = 573)

	CD patients	%	Controls	%	OR	p
IL-10R						
2	425	95.3	552	96.3	0.77	0.406
3	165	37.0	198	34.5	1.11	0.420
4	9	2.0	19	3.3	0.60	0.209
IL-10G						
7	21	4.7	16	2.8	1.72	0.105
8	36	8.1	34	5.9	1.39	0.181
9	248	55.6	306	53.4	1.09	0.484
10	65	14.6	75	13.1	1.13	0.494
11	107	24.0	140	24.4	0.98	0.870
12	54	12.1	63	11.0	1.12	0.580
13	170	38.1	263	46.0	0.73	0.013
14	81	18.2	81	14.1	1.35	0.081
15	9	2.0	18	3.1	0.64	0.268

**Table 2:** Carrier frequencies of promoter SNPs haplotypes in CD patients (n = 425) and controls (n = 529)

	CD patients	%	Controls	%	OR	p
ACC	239	56.24	287	54.25	1.08	0.541
ATA	186	43.76	235	44.42	0.97	0.839
GCC	275	64.71	332	62.76	1.04	0.535

Extended haplotypes including the *IL-10 *microsatellites and the promoter SNPs studied were also established. Haplotype frequencies were compared between patients and controls and also by means of a TDT in a familial study, but no extended haplotypes were found to be associated with or linked to CD (data not shown). Haplotypic frequencies obtained are similar to the previously described [18].

The strong influence of HLA-DQA1*0501 and DQB1*02 (HLA-DQ2) on the development of celiac disease could be masking a potentially weak effect of *IL-10 *polymorphisms on CD. To try to circumvent this problem, we performed a stratified analysis, comparing DQ2-positive with DQ2-negative patients (9%) and with controls, but no significant differences were observed among those groups (data not shown).

## Discussion

In this study we have performed a case-control and a familial study to assess the involvement of *IL-10 *polymorphisms in celiac disease. The results obtained seem to indicate that *IL-10 *is not an important susceptibility genetic factor for CD. This would seem an unexpected result since previous studies had revealed altered cytokine levels in several inflammatory conditions. Nevertheless, the most consistent finding in CD patients has been an increased production of interferon gamma, while IL-10 levels tend to remain at normal levels, although contradictory results do exist [19-21]. It has been described occasionally an increase in IL-10 mRNA levels in the intestine of celiac disease patients, but it is possible that this is a counter-regulatory mechanism triggered by the disease, and not the cause of the pathology [21]. This interpretation is supported by the fact that after gluten removal from the diet, IL-10 levels return to normal levels [19].

There have been few studies of IL-10 polymorphisms with celiac disease, mostly with negative results [13,14]. A recent report in Swedish population [4] describes an increase in homozygous -1082 AA individuals in patients compared to controls; we were not able to detect this effect, although our sample size was significantly higher. A recent study also in a Spanish population found an increase in the frequency of the GCC promoter haplotype when DQ2-positive patients were compared to DQ2-negative patients [22]. However, despite our considerable sample size, we have not been able to replicate that finding (41% in DQ2-positive patients vs. 42% in DQ2 negative patients). A recent Italian report has described a combined effect of -1082 and TNF-308A polymorphism in celiac disease [23], but although in our population an effect of the TNF-308A polymorphism does exist [24], we do not observe an interaction between both genes.

Albeit negative, our study extends considerably previous reports in terms of sample size, genetic markers included and familial haplotype analyses. Besides, our negative result does not preclude a relevant role of IL-10 in the pathogenesis of the disease. It may happen that the functional variation ascribed to the polymorphisms we have studied is not important in the specific cell type that produces IL-10 in the intestinal epithelium, which has been identified variously as Th2, Th3 or T regulatory cells. Not uncommonly, the expression of the same gene is under control of distinct regulatory elements in different cell types [25,26]. The possibility remains that the genetic variations we have studied, and that are associated with IL-10 production *in vitro *using peripheral blood leukocytes stimulated with LPS [7] or with ConA [27], may not replicate adequately the situation found in the intestinal mucosa. It has been described how gene variations in the *IL-10 *gene promoter affect IL-10 production depending on the stimulation used [28]. Additional polymorphisms, like those located in the distal promoter region of the *IL-10 *gene, may regulate the transcription rate of this cytokine in the mucosa. In order to not confine ourselves to the study of the already-known promoter polymorphism, we have included microsatellites in our research. The highly polymorphic IL-10R and, especially, IL-10G microsatellites are good genetic markers of the whole extended promoter, and no association whatsoever has been found in the present study.

Although there may be not differences in the basal level of IL-10 in the population, it would nevertheless be important to keep in mind the possible relevance of this cytokine in Celiac Disease but probably as a consequence of other immunological processes related to CD and not as the origin of the disease.

## Conclusion

Interleukin-10 polymorphisms do not seem to be involved in celiac disease predisposition in the Spanish population.

## Abbreviations

CD: celiac disease.

IL-10: interleukin-10.

SNP: single nucleotide polymorphism.

PCR: polymerase chain reaction.

HLA: human leukocyte antigen.

Th1: T helper type 1.

RhIL-10: recombinant human IL-10.

EM algorithm: Expectation-Maximization algorithm.

TDT: Transmission Disequilibrium Test.

LPS: lipopolysaccharide.

## Competing interests

The author(s) declare that they have no competing interests.

## Authors' contributions

CN supervised the genetic analysis and drafted the manuscript. DA and JV carried out the genetic analysis of the samples and participated in the statistical analysis. IP, CM and MFA participated in the collection of samples and participated in the design and coordination of the study. EGC participated in the design of the study and revised critically the manuscript. EU drafted the manuscript and revised it critically. AM conceived the study, participated in its design and coordination and revised critically the manuscript.

## Pre-publication history

The pre-publication history for this paper can be accessed here:


